# Surfactant-Assisted Regulation of WS_2_/Tourmaline Microstructures for Excellent Photocatalytic Performance

**DOI:** 10.3390/molecules29194555

**Published:** 2024-09-25

**Authors:** Xianku Wang, Kaibin Cui, Yuqin Zhao, Ming Hao, Liang Bian, Mingming Wang, Fei Wang

**Affiliations:** 1 Key Laboratory of Special Functional Materials for Ecological Environment and Information, Hebei University of Technology, Ministry of Education, Tianjin 300130, China; 2 Institute of Power Source and Ecomaterials Science, Hebei University of Technology, Tianjin 300130, China; 3 State Key Laboratory of Environment-Friendly Energy Materials, Southwest University of Science and Technology, Mianyang 621010, China; 4 School of Environment and Resource, Southwest University of Science and Technology, Mianyang 621010, China

**Keywords:** tungsten disulfide, surfactant, tourmaline, photocatalysis

## Abstract

The controllable electrical and optical properties of two-dimensional tungsten disulfide (WS_2_) attracted much attention in photocatalysis, but commercial development has been severely restricted by their restacking properties. Surfactant-assisted synthesis techniques can be considered as an effective option to break this bottleneck. In this work, the effect of surfactants including sodium dodecylbenzene sulfonate (SDBS), hexadecyltrimethylammonium bromide (CTAB), and polyvinylpyrrolidone (PVP) on the microstructure of WS_2_/tourmaline composites prepared by coupled hydrothermal and calcination methods was explored. The WS_2_ nanosheets were uniformly deposited on the tourmaline surface with the assistance of 1.0 mmol/L SDBS. Meanwhile, WS_2_/Tour-SDBS exhibited the highest rhodamine B (RhB) degradation activity, which was 1.8 and 2.3 times higher than that of photocatalysts prepared with CTAB and PVP under the same conditions, respectively. This study provides a new tactic for the fabrication of high-performance WS_2_-based composites.

## 1. Introduction

With the development of industrialization and urbanization, a large amount of organic dye is discharged into the water, which not only destroys the ecological environment but also seriously threatens human health [1]. Photocatalytic degradation is a common treatment method for organic dye wastewater [2,3], so the selection of catalyst materials is particularly important. In recent years, semiconductor photocatalysts have received extensive attention from researchers due to their non-toxicity and low-cost photocatalytic performance [4]. Tungsten disulfide, a crucial player in the transition metal disulfide (TMD) family, is a two-dimensional layered material with a similar structure to graphene. The basic structural unit of WS_2_ is the S-W-S interlayer, formed by two layers of S atoms covalently bonded to a layer of W atoms, with adjacent interlayers linked together by van der Waals forces [5]. WS_2_ has been widely used as a photocatalyst [6,7] and an electrocatalyst [8,9] due to its controllable optical and electrical properties. However, the inevitable stacking and agglomeration caused by the high specific surface energy result in the reduction of surface active sites, which leads to a decrease in catalytic efficiency. Several tactics were designed by researchers to address this difficulty, such as adopting a carrier [10] and atomic layer deposition [11,12]. Although these methods have demonstrated some achievement in improving the dispersion of WS_2_ nanostructures, the complex processes and high costs have limited commercial development.

As one of the amphiphilic compounds, surfactants generally consist of a hydrophilic head polar group and a hydrophobic tail of non-polar hydrocarbon chains. Surfactants can significantly reduce interfacial surface tension and free energy to inhibit particle agglomeration even at very low concentrations [13], and assisted synthesis techniques are an ideal option for improving the physicochemical properties of nanostructured materials, which are commonly used to optimize WS_2_-based composites [14]. Tourmaline, a natural mineral that is low-cost and environmentally friendly, is widely employed as a carrier material due to its excellent adsorption properties and large specific surface area [15]. In addition, the spontaneous polarization and far-infrared radiation properties promote atomic nucleation and growth by enhancing thermal vibrations and reducing diffusion activation energy [16]. In this work, WS_2_/tourmaline composites were fabricated for the photocatalytic degradation of organic dyes by coupling hydrothermal and calcination methods with the assistance of surfactants, including CTAB, PVP, and SDBS. The effects of surfactant type and concentration on the microstructure and photocatalytic performance of the composites were explored. This study provides a new scheme for the surfactant-assisted preparation of high-quality WS_2_-based photocatalysts.

## 2. Results and Discussion

**Figure 1:**
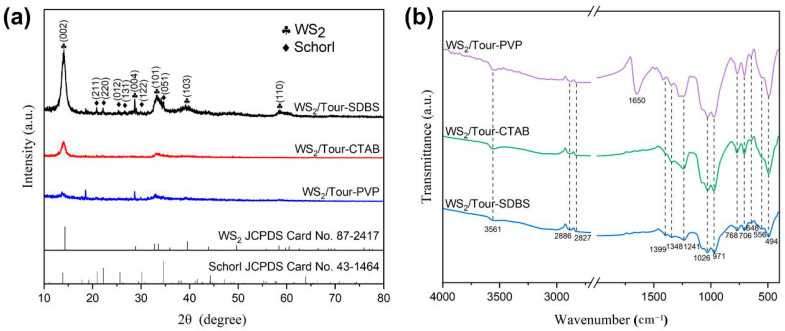
(**a**) X-ray diffraction patterns and (**b**) FTIR spectra of the samples synthesized with different surfactants.

The component crystalline phase information of the samples prepared with different types of surfactants (1.0 mmol/L) was characterized by X-ray diffraction measurements (Figure 1a). It can be clearly seen from WS_2_/Tour-SDBS that the diffraction peaks located at 14.1°, 28.7°, 33.4°, 39.6°, and 58.7° correspond to the (002), (004), (101), (103), and (110) planes of WS_2_, respectively (JCPDS No. 87-2417). The peaks with 2θ values of 20.9°, 22.2°, 25.6°, 26.3°, 30.2°, and 34.7° matched well with the (211), (220), (012), (131), (122), and (051) planes of tourmaline, respectively (JCPDS No. 43-1464). The planes in WS_2_/Tour-SDBS show a high intensity of diffraction peaks, indicating excellent crystallinity of WS_2_ with the assistance of SDBS. However, WS_2_ exhibited deteriorated crystallinity in samples prepared by similar processing coupled with PVP or CTAB.

The functional groups and chemical bonds on the composite surface were identified by FTIR analysis (Figure 1b). For tourmaline, the three peaks appearing at 706 cm^−1^, 768 cm^−1^, and 1026 cm^−1^ were generated by Si-O-Si stretching vibrations; the octahedral cation M-O was detected at 494 cm^−1^ and 648 cm^−1^; the peaks obtained at 1241 cm^−1^ and 1348 cm^−1^ were both attributed to the antisymmetric B-O; and the peaks at 971 cm^−1^ and 3561 cm^−1^ corresponded to Si-O-Al and -OH, respectively [17,18]. Meanwhile, the absorption peaks located at 556 cm^−1^ and 1399 cm^−1^ can be attributed to the W-S stretching vibration and hydroxyl stretching deformation in WS_2_ [19]. In addition, the peaks at 2827 cm^−1^ and 2886 cm^−1^ for the three samples were attributed to C-H bond vibrations [20], while the strong and sharp peak of WS_2_/Tour-PVP at 1650 cm^−1^ corresponded to the stretching of the C-O bond [21], which was caused by the residual surfactant. Based on the above analysis, it can be confirmed that the presence of surfactants has almost no effect on the surface groups of WS_2_ and tourmaline.

**Figure 2:**
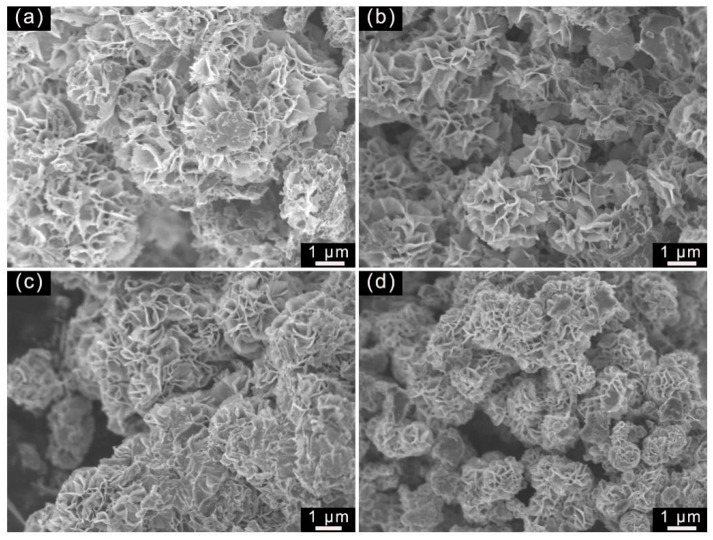
SEM images of the samples synthesized with different surfactants. (**a**) Control; (**b**) SDBS; (**c**) CTAB; and (**d**) PVP.

It is obvious that the different surfactants (1.0 mmol/L) used in the hydrothermal-calcination method can greatly influence the morphology of the as-fabricated samples. As shown in Figure 2a, the WS_2_/Tour composite consisted of WS_2_ nanosheets grown on the tourmaline surface, but with high agglomeration in some of these nanosheets. In the SDBS-assisted synthetic system, the WS_2_ nanosheets with smooth surfaces were homogeneously deposited on the tourmaline surface (Figure 2b). The porous structure of WS_2_/Tour-SDBS reduces the diffusion potential resistance of reactants [22] and facilitates the reflection and scattering of visible light between WS_2_ nanosheets, which improves the light trapping ability [23]. In contrast, the WS_2_ nanosheets became further dense on the tourmaline surface when CTAB was added to the precursor solution, forming clusters of large-size structures (Figure 2c). On the other hand, the non-ionic surfactant PVP modifies the crystalline anisotropic growth by weakening the van der Waals forces and thus has a limited effect on the morphology of the composites [24]. WS_2_/Tour-PVP exhibits a structure with microspheres of approximately 1 μm diameter adhered to each other (Figure 2d), so the existence of PVP is disadvantageous to improving the dispersibility of WS_2_ nanosheets.

**Figure 3:**
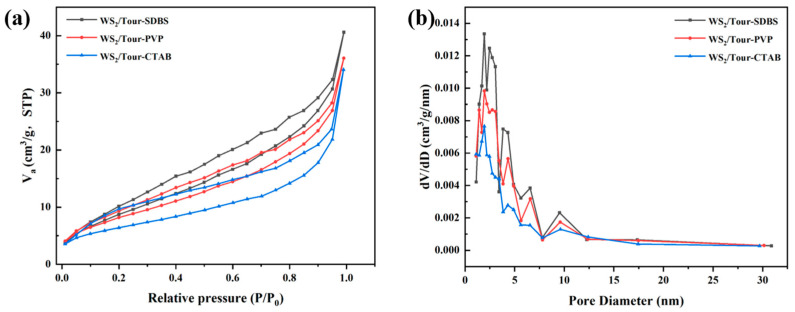
(**a**) N_2_ adsorption–desorption curves and (**b**) pore size distributions at the sample site.

As can be seen from Figure 3a, all the samples belong to type IV isotherms, indicating that porous sublayer adsorption occurred. Moreover, due to the generation of capillary condensation, an obvious hysteresis phenomenon can be observed, showing a H3-type hysteresis loop on the adsorption isotherm, indicating that all these materials have a typical mesoporous structure. Compared with other samples, the specific surface area and pore volume of WS_2_/Tour-SDBS were large (Table 1), and the larger specific surface area and pore volume further improved the light trapping ability of the catalysts and increased the number of surface active sites, which promoted the generation of active radicals. In addition, the improved pore structure accelerated the diffusion of pollutants and active radicals during the photocatalytic degradation process, favoring the enhancement of photocatalytic activity, which was closely related to the uniform dispersion of WS_2_ nanosheets on the tourmaline surface.

**Figure 4:**
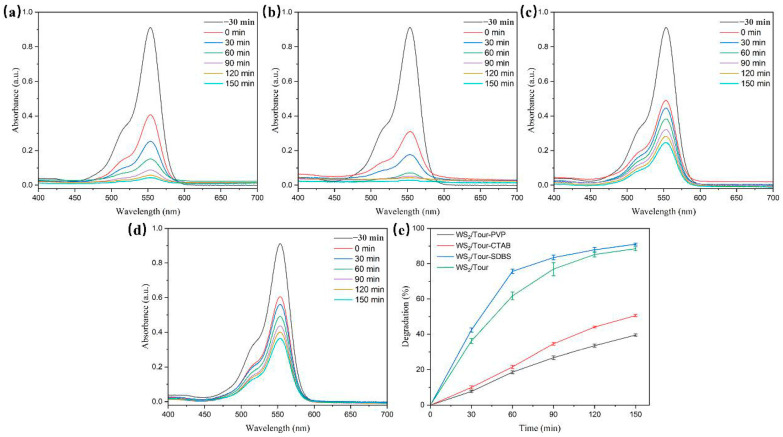
(**a**–**d**) Time dependence of the UV–vis absorption spectra in photodegradation of RhB on WS_2_/tourmaline prepared without surfactant (**a**), with SDBS (**b**), CTAB (**c**), and PVP (**d**). (**e**) Photocatalytic performance for as-prepared composites toward RhB degradation.

The photocatalytic performance was assessed by monitoring the absorbance of the RhB solution, as shown in Figure 4. The initial absorbance values at 553 nm for all catalysts decreased with prolonged visible light radiation, indicating that RhB was continuously being degraded. In comparison with samples prepared under other conditions, the homogeneous growth of WS_2_ nanosheets on the tourmaline surface with the assistance of SDBS resulted in a decrease in grain size and an increase in specific surface area, which in turn exposed sufficient photocatalytic active sites. WS_2_/Tour-SDBS exhibited the maximum degradation rate after 150 min of photocatalytic reaction, with approximately 91.1% of RhB removed, while WS_2_/Tour, WS_2_/Tour-CTAB, and WS_2_/Tour-PVP showed photocatalytic degradation rates of 89.4%, 50.6%, and 39.6% for RhB, respectively (Table 2). Based on the above results, the anionic surfactant SDBS was selected as the optimum surfactant.

**Figure 5:**
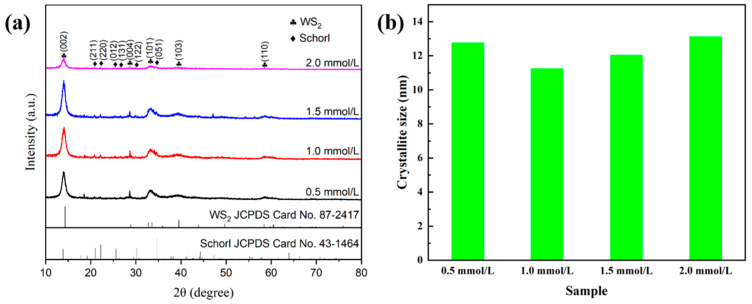
(**a**) X-ray diffraction patterns and (**b**) crystalline size of the samples synthesized with different concentrations of SDBS.

As shown in Figure 5a, the X-ray diffraction peaks of the samples synthesized at different concentrations of SDBS perfectly matched with WS_2_ (JCPDS No. 87-2417) and tourmaline (JCPDS No. 43-1464), indicating that changes in SDBS concentration have little effect on the phase composition. The diffraction peaks in the (002) and (101) planes of WS_2_ become sharper with increasing SDBS concentration, and the higher intensities show excellent crystallinity. However, the diffraction peaks could only be observed at 2θ values of 14.1°, 33.4°, and 34.7° when the SDBS concentration reached 2.0 mmol/L, and the peak intensity decreased significantly. This can be attributed to the fact that the SDBS concentration far exceeds the critical micelle concentration (cmc, 1.2 mmol/L) [25], resulting in an altered growth pattern of WS_2_ lamellae. In addition, the crystallite sizes of different samples were also calculated according to Scheller’s formula, and it was found that the crystallite size when the concentration of SDBS was 1.0 mmol/L (11.24 nm) was smaller than that of the samples with no added SDBS (about 11.68 nm), while the samples made under the other SDBS concentration conditions all had crystallite sizes larger than 11.68 nm (Figure 5b).

**Figure 6:**
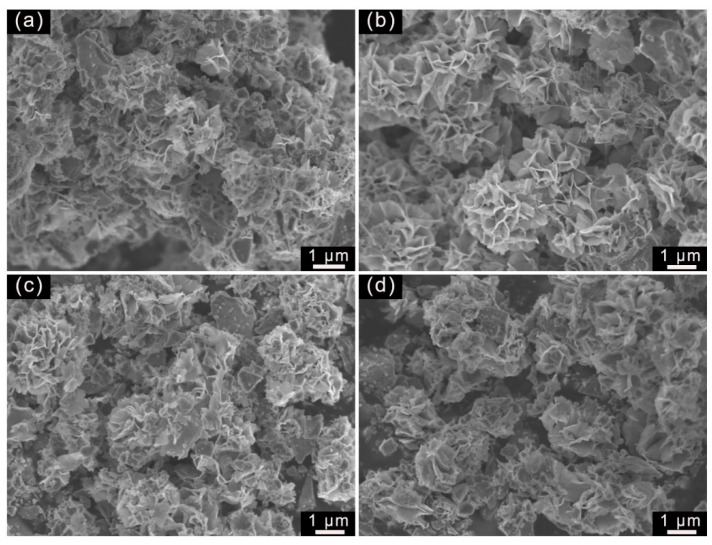
SEM images of the samples synthesized with different concentrations of SDBS: (**a**) 0.5 mmol/L; (**b**) 1.0 mmol/L; (**c**) 1.5 mmol/L; and (**d**) 2.0 mmol/L.

The morphology of the samples prepared with different concentrations of SDBS is revealed in Figure 6. It can be clearly seen that the WS_2_ was haphazardly dispersed on the tourmaline surface in the form of irregular nanosheets and nanoparticles when the SDBS concentration was 0.5 mmol/L. As the SDBS concentration is well below cmc, the SDBS molecules adhere to the WS_2_ surface and form a surfactant film with the aqueous solution, which inhibits crystal nucleation [26]. However, the solution surface tension weakens with increasing SDBS concentration, which reduces the inhibitory effect on crystal growth. The uniformly sized WS_2_ nanosheets cross-linked with each other on the tourmaline surface to form a regular porous structure as the SDBS concentration increased to 1.0 mmol/L. Nevertheless, the number of WS_2_ nanosheets gradually decreased with further increases in SDBS addition, especially when the SDBS molecules formed micelles at 2.0 mmol/L, which modified the lamellar structure of WS_2_.

**Figure 7:**
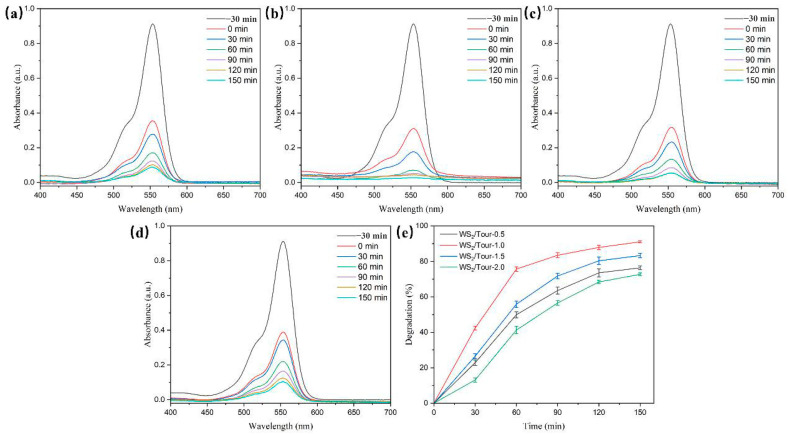
Time dependence of the UV–vis absorption spectra in photodegradation of RhB on WS_2_/tourmaline prepared with different concentrations of SDBS: (**a**) 0.5 mmol/L; (**b**) 1.0 mmol/L; (**c**) 1.5 mmol/L; and (**d**) 2.0 mmol/L. (**e**) Photocatalytic performance for as-prepared composites toward RhB degradation.

The photocatalytic performance of the catalyst for RhB degradation can be used as important evidence for obtaining the optimum concentration of SDBS. As the concentration of SDBS increased, the photocatalytic activity of the samples increased and then decreased, with the 1.0 mmol/L sample exhibiting the highest RhB degradation rate (Figure 7). The improved dispersion of WS_2_ nanosheets increased the contact area between the surface active site and the RhB molecules, with approximately 91.1% of the RhB being removed after 150 min of visible light irradiation. In contrast, the degradation rate of RhB solution was 76.4%, 83.3%, and 72.7% for the samples with SDBS concentrations of 0.5 mmol/L, 1.5 mmol/L, and 2.0 mmol/L, respectively (Table 3). Therefore, the optimum SDBS concentration can be determined to be 1.0 mmol/L.

**Figure 8:**
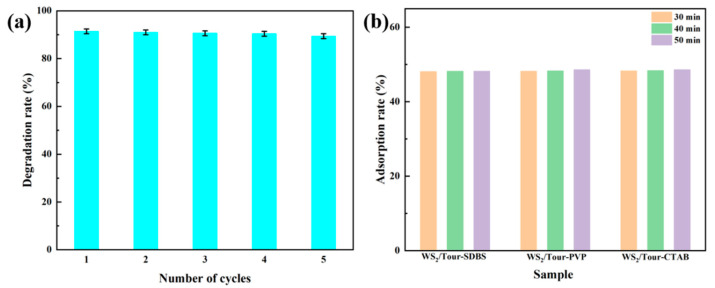
(**a**) Cyclic regeneration assay for RhB degradation by WS_2_/Tour-SDBS. (**b**) Diagram of adsorption properties of catalysts during dark reactions.

In addition, we also carried out cyclic regeneration experiments on the adsorbent (Figure 8a), and it can be seen that WS_2_/Tour-SDBS still has a high degradation rate of RhB (89.2%) after five cycles. Interestingly, all three composites have reached the adsorption–desorption equilibrium at the end of the 0.5 h dark reaction, and the adsorption rate is around 50% (Figure 8b). Furthermore, we compared some recently reported WS_2_-based nanocomposites for photocatalytic degradation of RhB and found that WS_2_/Tour-SDBS showed the best degradation efficiency for RhB (Table 4), showing very promising photocatalytic activity.

**Figure 9:**
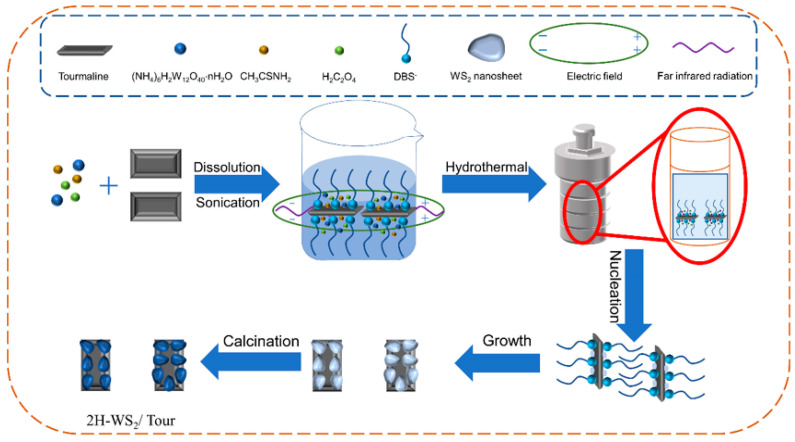
Growth mechanism of WS_2_/Tour with SDBS as a soft template.

According to the characterization analysis and photocatalytic activity test, the growth mechanism for the microstructure of WS_2_/Tour composites by anionic surfactant SDBS is proposed (Figure 9). The SDBS dissolved in water ionizes the dodecylbenzene sulfonate group (DBS^−^). The polar head of DBS^−^ was perpendicular to the tourmaline surface under the polarizing electric field [27] and constitutes a spatial site resistance as a soft template channel for WS_2_ crystal nuclei growth. The non-polar tail of DBS^−^ will bend under hydrophobic repulsion when the growth template encounters other templates, and adjacent tourmaline forms an isometric-oriented growth morphology [28], resulting in a significantly reduced stacking of WS_2_ nanosheets.

**Table 4:** Comparison of photodegradation activity of RhB with WS_2_-based materials.

Material	Catalyst Loading	RhB Concentration	Degradation Rate	Ref.
WS_2_	10 mg	100 mL of 10 mg/L	65.7%	[29]
WS_2_/BiOCl	20 mg	100 mL of 20 mg/L	80.1%	[30]
WS_2_/TiO_2_	20 mg	100 mL of 20 mg/L	86.1%	[31]
WS_2_/AgI	50 mg	150 mL of 10 mg/L	91.2%	[32]
WS_2_/MoS_2_	50 mg	50 mL of 10 mg/L	93%	[33]
WS_2_/Bi_2_MoO_6_	50 mg	50 mL of 10 mg/L	95%	[34]
WS_2_/Tour-SDBS	20 mg	100 mL of 20 mg/L	91.1%	This study

**Figure 10:**
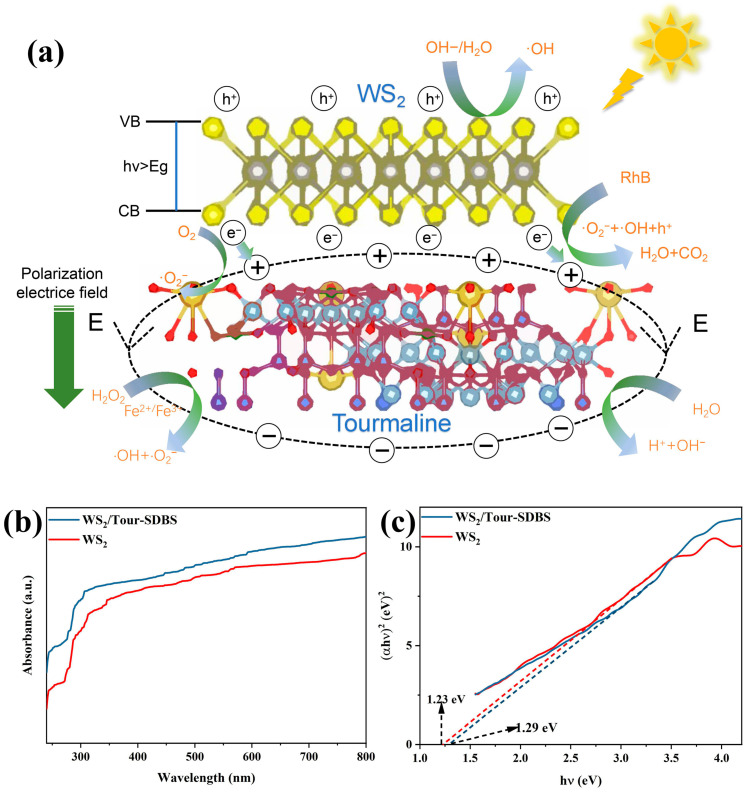
(**a**) Schematic diagram of the synergy between WS_2_ and tourmaline in RhB photocatalytic degradation. (**b**) UV–vis diffuse reflectance spectra of WS_2_/Tour-SDBS and WS_2_; (**c**) the corresponding plot of (αhν)^2^ versus hν for WS_2_/Tour-SDBS and WS_2_.

Based on the above results, the mechanism of photocatalytic degradation of RhB by WS_2_/tourmaline composites is proposed (Figure 10a). First, under visible light irradiation, electrons in the valence band (VB) of WS_2_ are excited to the conduction band (CB) and form corresponding holes (Equation (2)). Tourmaline has the effect of polarizing water molecules to produce OH^−^ (Equation (3)) [35], and the holes in turn oxidize OH^−^ to ·OH (Equation (4)). Second, the strong electrostatic field on the surface of tourmaline induces the directional transfer of electrons in the WS_2_ conduction band (Equation (5)) [36], which not only effectively reduces the complexation of electron–hole pairs but also reacts with dissolved oxygen in water to produce ·O_2_^−^ (Equation (6)). Third, the redox reaction can generate the intermediate product H_2_O_2_. Ferrous ions released from tourmaline can catalyze the formation of ·OH and ·O_2_^−^ (Equation (7)) from H_2_O_2_ [37]. Finally, h^+^, ·OH, and ·O_2_^−^ all react with RhB to form CO_2_ and H_2_O (Equation (8)). The chemical equations of the degradation process are as follows:

WS_2_ + hν → WS_2_ (e^−^ _CB_) + WS_2_ (h^+^ _VB_) (1)

H_2_O → H^+^ + OH^−^ (2)

h^+^ + OH^−^/H_2_O → ·OH (3)

WS_2_ (e^−^ _CB_) + Tourmaline → WS_2_ + Tourmaline (e^−^) (4)

Tourmaline (e^−^)/WS_2_ (e^−^ _CB_) + O_2_ → ·O_2_^−^+ WS_2_ + Tourmaline (5)

Tourmaline + H_2_O_2_ → ·OH + ·O_2_^−^ (6)

·O_2_^−^/·OH/h^+^ + RhB → CO_2_ + H_2_O (7)

To investigate the light absorption properties of the samples, they were tested by UV–vis diffuse reflectance spectroscopy. The UV–visible diffuse reflectance spectra of WS_2_/Tour-SDBS and WS_2_ are shown in Figure 10b. From Figure 10b, it can be seen that WS_2_ has a weak ability to absorb light, and the surfactant can improve the light absorption performance of WS_2_. After loading tungsten disulfide on the tourmaline surface and activation using SDBS, the light absorption performance of WS_2_/Tour-SDBS in the full spectral band was significantly improved. However, the light absorption performance of WS_2_/Tour-SDBS was slightly higher than that of WS_2_, which was mainly due to the weaker light absorption ability of tourmaline. Based on the UV-DRS results, Tauc’s formula was utilized to make a graph, and the intersection of the tangent line of its curve with the X-axis was the band gap energy of the sample. The results are shown in Figure 10c. As can be seen from the figure, the band gap energies of WS_2_/Tour-SDBS and WS_2_ are 1.29 eV and 1.23 eV, respectively, and the band gap energy of WS_2_/Tour-SDBS is slightly increased, which may be attributed to the fact that the WS_2_ in the composite material has a higher number of layers. Taken together, WS_2_ is the active component in the photocatalytic process, SDBS has the best optimization effect on the microstructure of WS_2_/tourmaline, and the composites still have good light absorption properties.

## 3. Experimental

### 3.1. Materials

Tourmaline (composition: SiO_2_ 33.46 wt.%, Al_2_O_3_ 29.88 wt.%, Fe_2_O_3_ 8.51 wt.%, B_2_O_3_ 7.96 wt.%, CaO 6.86 wt.%, FeO 3.83 wt.%, and MgO 3.55 wt.%) was obtained from HY Technology Co., Ltd. (London, UK). The ammonium metatungstate ((NH_4_)_6_H_2_W_12_O_40_·nH_2_O) was provided by Macklin (Shanghai, China). Thioacetamide (CH_3_CSNH_2_), oxalate acid (H_2_C_2_O_4_), and RhB were purchased from Kewei Chemical Group Co., Ltd. (Zibo, China). Hexadecyltrimethylammonium bromide (CTAB, C_19_H_42_BrN), sodium dodecyl benzene sulfonate (SDBS, C_18_H_29_NaO_3_S), and polyvinyl pyrrolidone (PVP, (C_6_H_9_NO)_n_) were provided by Tianjin Damao Chemical Reagent Group Co., Ltd. (Tianjin, China). All chemical reagents were used as received. Deionized (DI) water was used in all experiments.

### 3.2. Preparation of WS_2_/Tourmaline Composites

For a typical preparation procedure, 0.23 g of tourmaline powder was dispersed in 50 mL of deionized water and sonicated for 0.5 h. Subsequently, 0.89 g (3.4 mmol) of (NH_4_)_6_H_2_W_12_O_40_·nH_2_O, 1 g (13.3 mmol) of CH_3_CSNH_2_, and 0.68 g (7.5 mmol) of H_2_C_2_O_4_ were mixed in the above slurry and stirred magnetically for 0.5 h. Then, an appropriate dosage (0.5, 1.0, 1.5, and 2 mmol/L) of one surfactant (SDBS, CTAB, and PVP, respectively) was added to the above suspension, and stirring was continued for 10 min. After that, the final solution was directly transferred into a 100 mL para-polyphenylene stainless steel autoclave and maintained at 220 °C for 24 h. After hydrothermal reaction, the black samples were obtained by filtration, washing several times with deionized water, and drying at 60 °C for 12 h in a vacuum oven. Finally, the hydrothermal products were placed in a tube furnace under an H_2_/N_2_ atmosphere and kept at 300 °C for 3 h. After the above process, the samples synthesized with SDBS, CTAB, and PVP were named WS_2_/Tour-SDBS, WS_2_/Tour-CTAB, and WS_2_/Tour-PVP, respectively. As the control sample, WS_2_/Tour was synthesized using the same process without the assistance of surfactants.

### 3.3. Characterization

The crystal structures of the as-fabricated samples were examined on a Smartlab 9 kW X-ray instrument (JEOL, Tokyo, Japan) with Cu Kα radiation over a 2θ range between 10° and 80°. Infrared radiation spectra were recorded in transmission mode from 400 to 4000 cm^−1^ on a Fourier-transform infrared (FTIR) spectrometer (Bruker Co., Ltd., Billerica, MA, USA). The morphologies of the as-fabricated samples were observed with a 7610F scanning electron microscope (JEOL, Osaka, Japan) at an accelerating voltage of 30 kV. The specific surface areas and pore structures of the samples were measured by a Quantachrome autosorb-iQ_2_ analyzer (autosorb-IQ_2_, Quantachrome, Boynton Beach, FL, USA).

### 3.4. Photocatalytic Tests

The photocatalytic experiments were carried out under atmospheric pressure and ambient conditions at room temperature, and the photocatalytic activity of the catalysts was tested using RhB as the target substrate, with light absorption at 553 nm as the predominant wavelength. For a typical experimental process, a 20 mg powder sample was added to 100 mL of a 20 mg/L RhB solution, and the suspension was vigorously stirred for 0.5 h under dark conditions to ensure the establishment of an adsorption–desorption equilibrium. Next, the above suspension was subjected to irradiation by 500 W xenon lamp manufactured by Shanghai Xebe Technology Co. (Shanghai, China) equipped with a 420 nm UV cut-off filter and kept stirred. At 0.5 h intervals from the start of the test, approximately 6 mL of the suspension was extracted and centrifuged to achieve solid–liquid separation. The filtrates were analyzed by recording the UV–vis spectra by a Shimadzu UV-1900 spectrophotometer manufactured by Shimadzu Corporation, Kyoto, Japan, and the photocatalytic degradation efficiency was calculated by Equation (1), where D denotes the photodegradation efficiency of the sample for RhB, A_t_, and A_0_ denote the absorbance values at irradiation time t and initial irradiation time, respectively.

(8) $$D\;(\%)=(A_{0}-A_{t})/A_{0}\;\times \;100\%$$

## 4. Conclusions

In summary, the WS_2_/tourmaline composites were regulated by a surfactant-assisted hydrothermal-calcination method, and the influence of the surfactant type and concentration was investigated. The results showed that SDBS had the best effect on the optimization of the WS_2_/tourmaline microstructure, while the agglomeration of WS_2_ nanosheets was aggravated in the case of CTAB and PVP. The isometric orientation growth state with the assistance of SDBS resulted in a significant reduction in the accumulation of WS_2_ nanosheets on the tourmaline surface and thus exhibited superior photocatalytic activity toward RhB degradation due to the exposure of more photocatalytic sites on WS_2_ nanosheets. The sample fabricated with 1.0 mmol/L SDBS exhibited a RhB degradation rate of 91.1% under visible light irradiation for 150 min, which was much higher than the samples fabricated with CTAB (50.6%) and PVP (39.6%) under the same conditions. This work provides a new insight into surfactant-assisted regulation of WS_2_-based catalysts. 

## Figures and Tables

**Table 1:** Specific surface area, average pore size, and total pore volume of the samples.

Sample	Specific Surface Area (m^2^/g)	Average Pore Diameter (nm)	Total Pore Volume (cc/g)
WS_2_/Tour-SDBS	32.657	7.689	0.063
WS_2_/Tour-PVP	30.016	7.451	0.056
WS_2_/Tour-CTAB	22.955	9.198	0.053

**Table 2:** Photocatalytic parameters of different catalysts.

	30 min	60 min	90 min	120 min	150 min
WS2/Tour	36.4%	62.1%	75.1%	80.8%	89.4%
WS2/Tour-SDBS	41.2%	74.2%	81.0%	84.2%	91.1%
WS2/Tour-PVP	7.6%	18.2%	24.2%	29.3%	39.6%
WS2/Tour-CTAB	9.8%	20.2%	34.5%	46.4%	50.6%

**Table 3:** Photocatalytic parameters of catalysts with different SDBS additions.

	30 min	60 min	90 min	120 min	150 min
0.5 mmol/L	23.5%	49.8%	64.1%	73.8%	76.4%
1.0 mmol/L	41.2%	74.2%	81.0%	84.2%	91.1%
1.5 mmol/L	26.3%	54.7%	70.5%	78.2%	83.3%
2.0 mmol/L	14.2%	42.1%	56.2%	68.5%	72.7%

## Data Availability

No data was used for the research described in the article.
